# Commentary on “Nonpharmacological Methods to Reduce Pain During Active Labor in a Real-life Setting”

**DOI:** 10.1055/s-0043-1772470

**Published:** 2023-10-16

**Authors:** Roxana Knobel, Mariane de Oliveira Menezes, Carla Betina Andreucci

**Affiliations:** 1 Department of Obstetrics and Gynecology, Universidade Federal de Santa Catarina, Florianópolis, SC, Brazil; 2 Department of Gynecology and Obstetrics, Universidade Estadual Paulista “Júlio de Mesquita Filho,” Botucatu, SP, Brazil; 3 Department of Medicine, Universidade Federal de São Carlos, São Carlos, SP, Brazil

Dear Editor,

We have read with interest the article by Silva et al. 1 The study concluded that in a real-life setting there was no difference in the intensity of labor pain between patients who used nonpharmacological methods during the active phase of labor and those who did not. We appreciate the efforts of the research team, as their study undoubtedly contributes to enhance knowledge about labor and childbirth care. We find it crucial to highlight the widespread use of non-pharmacological methods for pain control in the institution in which the study was conducted, a highly recommended practice 2 that is not frequently available in Brazilian settings. 3

While we acknowledge the importance of their results, we would like to raise some concerns we believe are relevant for a more accurate understanding of their findings.

Measuring pain is challenging because pain is a personal, subjective, and multifaceted experience. Labor pain presents additional difficulties because of all the emotions and concerns involved in the process, the duration of the painful sensations (which can last several hours), and the increasing intensity of the pain during the progression of dilation and fetus descent through the maternal pelvis. 4 5

We have considered that one single evaluation using the Visual Analog Scale (VAS) after the delivery would not suffice to measure all multifaceted, variable, and usually long-lasting aspects of labor pain. In most of the studies using VAS included in the Cochrane Systematic Reviews, the tool was applied several times throughout the course of labor and/or before and after the intervention. 6 7 Additionally, although the VAS is widely used, more comprehensive instruments that enable multidimensional assessments of pain and the experience of the parturient are necessary. 5 Furthermore, regarding the interpretation of results, it would be important to consider not only pain, but also satisfaction, the perception of a safe environment, personal achievement, and situation control. 8 9

Moreover, there is a comparative issue that needs to be taken into consideration. The study compares groups that are not analogous in terms of the time of exposure to non-pharmacological methods. It is important to note that one group had little to no time to become familiar with the resources, and even use them, while the other group had a more extensive opportunity to become familiar with the pain control methods, and a longer active phase period.

Moreover, the abstract and conclusions of the aforementioned study may lead readers to believe that non-pharmacological methods are ineffective, when the measurement of pain was imprecise, and the groups were not comparable. We believe the misconceptions the conclusion produces might restrict women's access to non-pharmacological methods.

Overall, we believe the collected data depict an interesting quantitative analysis of non-pharmacological methods and factors associated with their use. In the abstract, we suggest the authors clarify that the method chosen to measure pain has limitations, and that the use of non-pharmacological methods to cope with pain should be encouraged.
